# The Uses of a Dual-Band Corrugated Circularly Polarized Horn Antenna for 5G Systems

**DOI:** 10.3390/mi13020289

**Published:** 2022-02-11

**Authors:** Chih-Kai Liu, Wei-Yuan Chiang, Pei-Zong Rao, Pei-Hsiu Hung, Shih-Hung Chen, Chiung-An Chen, Liang-Hung Wang, Patricia Angela R. Abu, Shih-Lun Chen

**Affiliations:** 1 Department of Physics, National Central University, Taoyuan City 320317, Taiwan; 110282001@cc.ncu.edu.tw (C.-K.L.); 102282004@cc.ncu.edu.tw (P.-H.H.); chensh@ncu.edu.tw (S.-H.C.); 2 National Synchrotron Radiation Research Center, Hsinchu City 30076, Taiwan; 3 Shenzhen Jaguar Wave Technology Co. Ltd., Shenzhen 518051, China; peizong.rao@jaguarwave.com; 4 Department of Electrical Engineering, Ming Chi University of Technology, New Taipei City 243303, Taiwan; 5 Department of Microelectronics, College of Physics and Information Engineering, Fuzhou University, Fuzhou City 350108, China; eetommy@fzu.edu.cn; 6 Department of Information Systems and Computer Science, Ateneo de Manila University, Quezon City 1108, Philippines; pabu@ateneo.edu; 7 Department of Electronic Engineering, Chung Yuan Christian University, Taoyuan City 320314, Taiwan

**Keywords:** circularly polarized antenna, corrugated antenna, dual-band, mm-wave band, 5G system

## Abstract

This paper presents the development of a wide-beam width, dual-band, omnidirectional antenna for the mm-wave band used in 5G communication systems for indoor coverage. The 5G indoor environment includes features of wide space and short range. Additionally, it needs to function well under a variety of circumstances in order to carry out its diverse set of network applications. The waveguide antenna has been designed to be small enough to meet the requirements of mm-wave band and utilizes a corrugated horn to produce a wide beam width. Additionally, it is small enough to integrate with 5G communication products and is easy to manufacture. This design is simple enough to have multi-feature antenna performance and is more useful for the femtocell repeater. The corrugated circularly polarized horn antenna has been designed for two frequency bands; namely, 26.5–30 GHz for the low band and 36–40 GHz for high band. The results of this study show that return-loss is better than 18 dB for both low and high band. The peak gain is 6.1 dBi for the low band and 8.7 dBi for the high band. The beam width is 105 degrees and 77 degrees for the low band and the high band, respectively. The axial ratio is less than 5.2 dB for both low and high band. Generally, traditional circularly polarized antennas cannot meet the requirements for broadband. The designs for the antennas proposed here can meet the requirements of FR2 bandwidths. This feature limits axial ratio performance. The measurement error in the current experiment comes from the high precision control on the size of the ridge.

## 1. Introduction

Millimeter-wave communication is a key technology for 5G mobile networks. The 5G standard extends the frequency spectrum to 28 GHz and 39 GHz in millimeter-wave band. This wireless system design will have benefits, such as high data rates and low latency, which will be utilized in new technological applications. However, there are several challenges in developing a 5G wireless system. For instance, atmospheric attenuation and high-path loss become critically high at millimeter-wave frequency bands. The 5G mm-wave networks have the advantages of ultra-high throughput and ultra-low latency, therefore offering a better experience on emerging platforms, such as health care, high-definition video streaming, high-definition gaming, and real-time virtual reality. However, compared to wireless transmission systems below 6 GHz which scatter and diffuse, the high-frequency millimeter-wave system has difficulty in covering broader indoor spaces. The 5G repeater system will play an important role in solving this issue of inadequate indoor coverage. It will amplify the signal from the outdoor base station and broadcast the signal to indoor environments. Additionally, plans for 5G will include various multi-signal paths and the mismatching of polarization. This paper proposes a novel design that possesses both circular polarization and wide beam width properties that perfectly match the 5G repeater system demand [1].

The application of circularly polarized antennas in communication originated in satellite communication. The polarization plane of a linearly polarized wave will be deflected and lead to a polarization mismatch due to the Faraday rotation effect [2,3], which is produced by electromagnetic waves passing through the ionosphere. The smaller the elevation angle of the antenna, the greater the impact. However, the Faraday rotation effect has almost no effect on the circularly polarized wave, which makes the circularly polarized antenna extremely important in satellite communications. Circular polarization antennas are also used in general communication systems to reduce polarization mismatch, increase signal coverage, and inhibit multipath errors [1]. The grooved waveguide, iris-loaded waveguide, septum polarizer, and ridge circular waveguide are the main design structures of circularly polarized antennas [4]. 

In the communication industry, the mm-wave 5G repeater system is the future [5]. The radiated power of the repeater system is about 10 dBm, and the antenna gain is 6 dBi. Based on the linked budget and path loss calculations [6], the signal power will cover about 5~10 m in an indoor environment. The axial ratio will be under 6 dB to mitigate polarization mismatch [7].

Previous research has studied the circular polarized horn antenna in millimeter-wave band. A low-cost circularly polarized horn antenna has a wide band (50–75 GHz) and low axial ratio (AR), as shown in [8]. Circularly polarized horn antennas exploiting open slotted end structures have a wide band (75–110 GHz) and low AR (< 3 dB) [9]. This seems to provide a solution, but fall short with regards to beam width coverage on 5G repeater antennae. Carlos and Fabiano presented a design for a broad beam width, with a 60 GHz band [10]. Corrugated structure antenna successfully achieved 112 degrees of antenna beam width at 55–62.5 GHz. It will overcome the lack of coverage indoors, but this design still struggles with various applications.

In this study, a dual-band circularly polarized horn antenna with broad beam width is presented to solve the issues of indoor coverage and other usage scenarios. Broad beam width can be achieved by using two different corrugated structures. The double ridged waveguide polarizer was used on the horn antenna so that it would have dual-band circularly polarization properties. To ascertain the performance of the proposed antenna, a prototype running at a 5G FR2 band was fabricated. The operation’s band frequencies were n257 and n260 [11]. This antenna design achieves 105 degrees of 3 dB beam width on low band and 77 degrees of 3 dB beam width on high band, with a return loss greater than 15 dB. The antenna axial ratio reached a 3~5 dB level. The antenna-gain measurement was 6.1 dBi at low band and 8.5 dBi at high band. 

**Figure 1:**
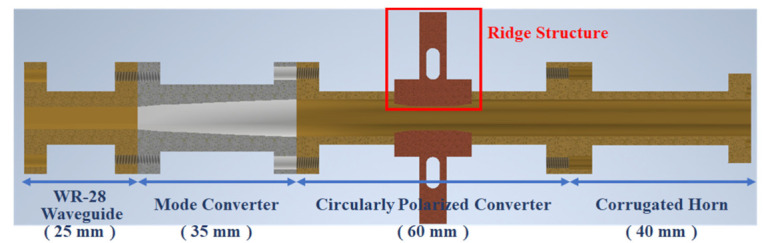
The cross-section view of the dual-band corrugated circularly polarized horn.

The proposed antenna was constructed by a mode converter, a circularly polarized converter, and a corrugated horn. In order to satisfy the space of mm-wave band operated conditions, the WR-28 rectangular waveguide was used to design the input port. The mode converter, the double-ridge polarization converter [4], and the corrugated horn are the main components of this proposed antenna. The double-ridge circular waveguide can be used to design a high performance circularly polarized converter. The mode converter is the adapter that transfer the circular waveguide to the standard WR-28 waveguide. The corrugated horn is designed to enhance the beam width. The total structure of the proposed antenna is illustrated in Figure 1, and it is suitable for femtocell repeater application.

## 2. Mode Converter and Circularly Polarized Converter Design

### 2.1. Mode Converter

**Figure 2:**
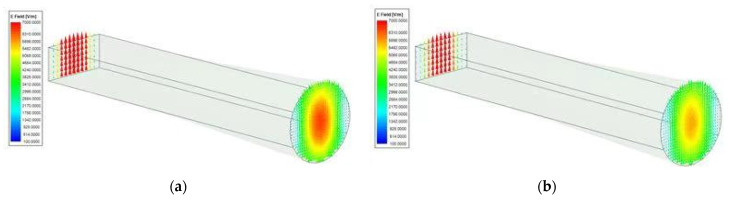
E field pattern of mode converter at: (**a**) 28 GHz and (**b**) 39 GHz. (Normalized to max value for red areas and minimum value for blue areas).

The input port of the mode converter is a standard WR-28 waveguide, with a TE_10_ transmission mode and its E-field type is shown in Figure 2. The output port is a circular waveguide with a radius of 4.2 mm with a TE_11_ transmission mode. The E-field pattern of the circular waveguide is shown in Figure 2 as well. Both modes are the fundamental mode of these types of waveguides. In order to make the mode conversion and transmission efficiency of the microwave signal between the two waveguides high, the length of the mode converter must be designed with a low insertion loss. The insertion loss of the proposed mode converter is less than 0.05 dB. The electric field pattern of this structure at 28 GHz and 39 GHz is illustrated in Figure 2a,b, respectively. The TE_10_ mode transmitted in the WR-28 waveguide can be smoothly converted into the TE_11_ mode of the circular waveguide with low insertion loss.

### 2.2. Circularly Polarized Converter

In the previous research results [4], it is found that the single ridge structure has a very good effect on narrow frequency applications. In the proposed design, double ridge structure is used since the single ridge structure cannot meet the design conditions at both the low-frequency band (26.5–30.0 GHz) and the high-frequency band (36.0–40.0 GHz). The ridge structure is designed to be tunable and this flexible design will benefit its use in a wide range of applications. 

**Figure 3:**
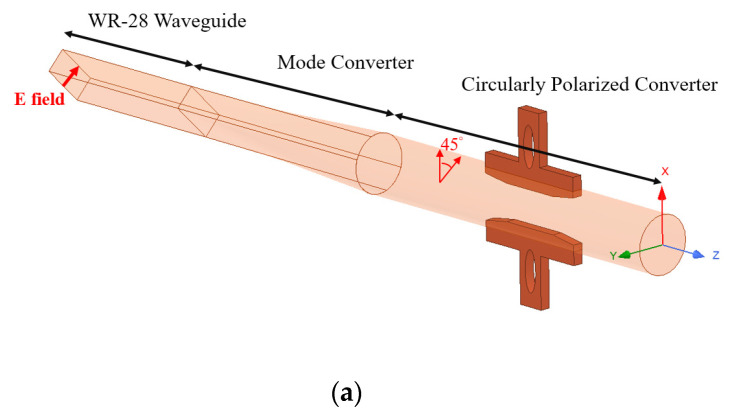

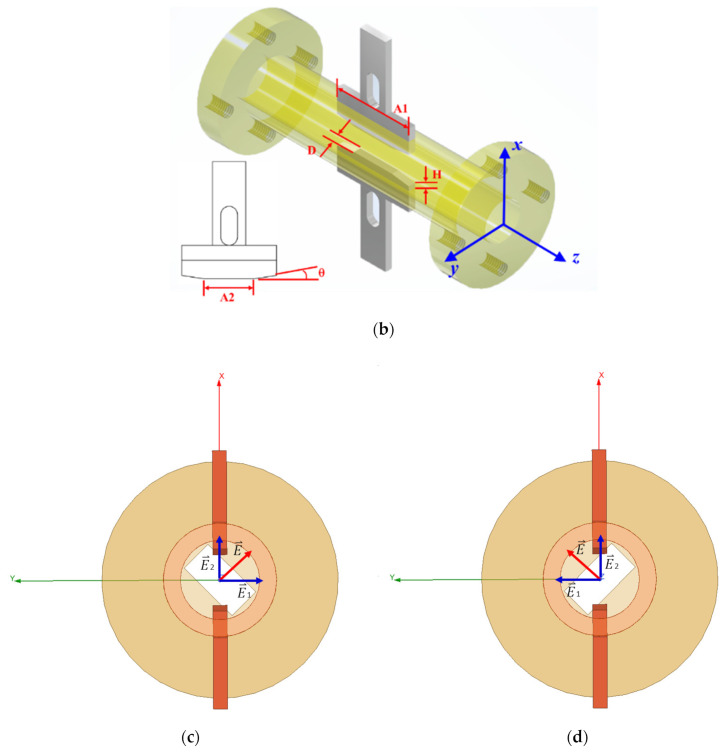
(**a**) Schematic of mode converter and circular polarization, (**b**) 3D structure of circular polarization converter, (**c**) decomposition of an incident linearly polarized TE_11_ wave-right hand circular polarization (RHCP) (this study), and (**d**) decomposition of an incident linearly polarized TE_11_ wave-left hand circular polarization (LHCP).

The schematic layout of the mode converter and circularly polarization converter is shown in Figure 3a while the 3D structure and parameters of the circular polarization converter are shown in Figure 3b. The ridged polarizer is located at a ±45° offset with respect to the E field axis, and an incident linearly polarized wave (TE_11_ mode), with polarization orientation which lies at the center between the x axis and y axis, is assumed, as shown in Figure 3c. 

A linearly polarized TE_11_ wave in circular waveguide can be decomposed into two equal-amplitude orthogonal linearly polarized TE_11_ waves, with a circularly polarized wave represented by the superposition of two equal-amplitude orthogonal TE_11_ waves which have 90° differential phase shifting between them [12]. In the polarization converter region, the propagation constants *β*_1_ and *β*_2_ of the E_1_ and E_2_ field are not the same because the cross section of circular waveguide is slightly perturbed by the ridged polarizer. E_2_ causes more phase delay than E_1_. This will make the antenna act as a right-hand circularly polarization antenna. Consequently, if the rectangular waveguide is rotated by 90°, a left-hand circularly polarization antenna will be created, as shown in Figure 3d. 

Ridged polarizer can be accomplished by choosing an appropriate height, width, and length for the ridged polarizer to attain a 90° phase difference at the circular waveguide output port. The theoretical phase difference can be estimated using the formula in Equation (1).

(1) $$\begin{array}{ll} \Delta \psi & =\psi_{1}-\psi_{2}=\left(-\beta_{1}\ell \right)-\left(-\beta_{2}\ell \right)=\left(\beta_{2}-\beta_{1}\right)\ell =2\pi \ell \left(\frac{1}{\lambda_{g2}}-\frac{1}{\lambda_{g1}}\right)\\ & =\frac{2\pi \ell }{c}\left(\sqrt{f^{2}-f_{c2}^{2}}-\sqrt{f^{2}-f_{c1}^{2}}\right) \end{array}$$

where Δψ is the phase difference of orthogonal TE_11_ mode, ℓ is the length of the ridged polarizer, $f_{c1}$ and $\;f_{c2}\;$ are the cutoff frequencies of E_1_ and E_2_, respectively.$\;f_{c1}\;$ and $\;f_{c2}\;$ can be determined by the transverse resonance technique [13].

**Figure 4:**
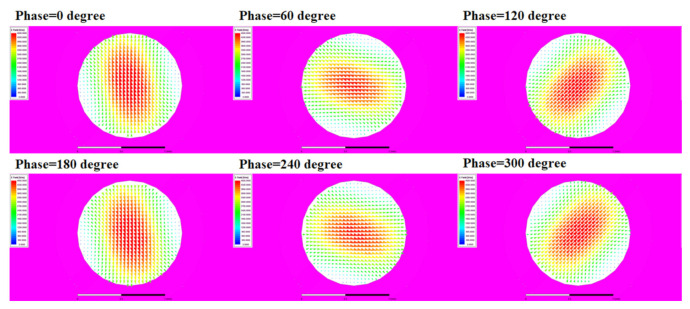
E field pattern of waveguide with different phases (at 28.5 GHz).

According to the formula, the phase of the electric field is affected by the length of A1. The amplitude of the electric field is compressed by the ridges that are controlled by the parameters H and D, while the return loss can be optimized by changing the angle θ of the ridge. The optimized parameters of the circularly polarized converter are listed in Table 1. Figure 4 presents the electric field versus phase change graphs at an operating frequency of 28.5 GHz. The circularly polarized wave can be produced by this circularly polarized converter.

## 3. Corrugated Antenna Design

**Figure 5:**
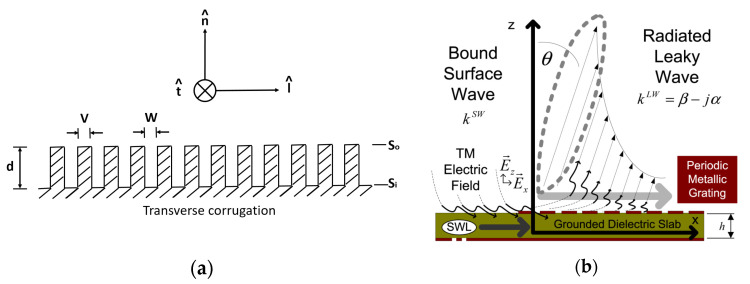
(**a**) Definition of soft surface and (**b**) leaky-wave antenna.

A corrugated structure has been a part of the horn antenna’s application for many decades. When the horn antenna radiates on the open end of the horn structure, the electromagnetic wave will creep on the metal surface to become surface wave. This will increase the side lobe of the far field pattern and decrease the antenna gain. Then, the corrugated structure will mitigate this phenomenon. Per-Simon Kildal [14] also defined the soft surface to describe the physics of surface wave on metal surfaces, as illustrated in Figure 5a. It pointed out that surface wave cannot propagate on soft surfaces by using a quarter wavelength depth of the corrugated structure. Furthermore, it stated that the resistance of wave incident direction is infinite and that the impedance of orthogonal direction on wave propagation is zero. This will cause the horn antenna to have a lower side lobe and a higher antenna gain.

**Figure 6:**
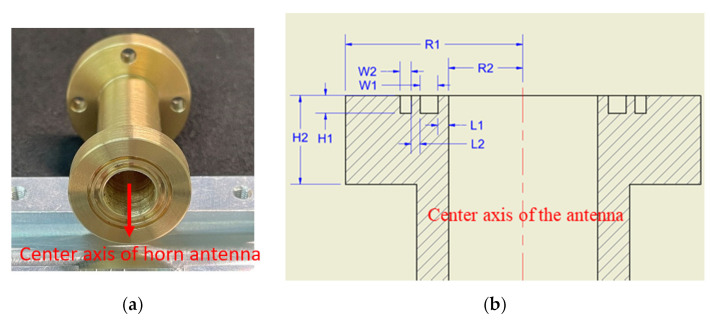
(**a**) Corrugated horn antenna, (**b**) parameter of cross section of detail corrugated antenna. R1 is the radius of the corrugated horn. W1 and W2 is the gap width of the corrugated horn, and H1 is the depth of the corrugated horn.

On the other hand, surface wave application has also been implemented to design a leaky-wave antenna, as shown in Figure 5b. Leaky wave is a characteristic of surface wave, and it can be observed as a signal source when propagating high dielectric substrate. It scatters far-field wave by using a periodic metal structure [15]. In Figure 6, a non-quarter wavelength of corrugated structure is applied to radiate far field by using the leaky-wave antenna theory. This, in effect, will contribute towards a broad beam width effect on the far field pattern of the horn antenna. Table 2 lists the optimized parameters of the corrugated horn antenna. Here, we use two different gap sizes W1 and W2 to control the low and high band beam width.

**Figure 7:**
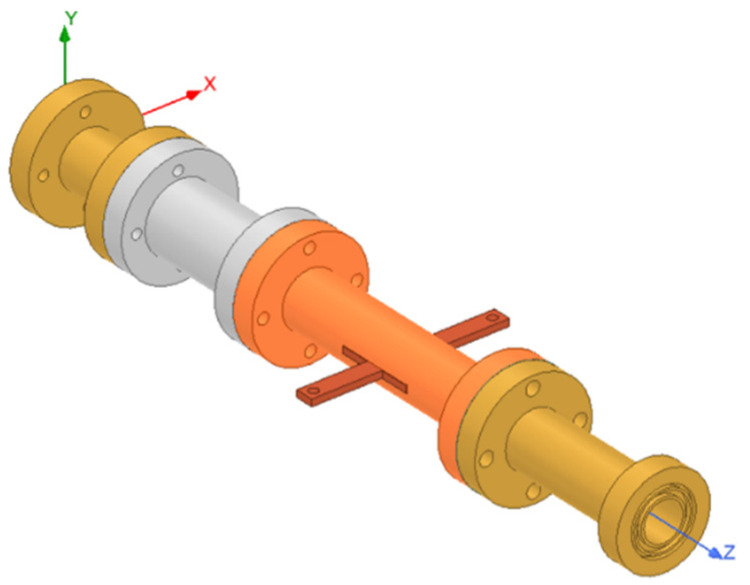
3D simulation coordinate of corrugated horn antenna.

**Figure 8:**
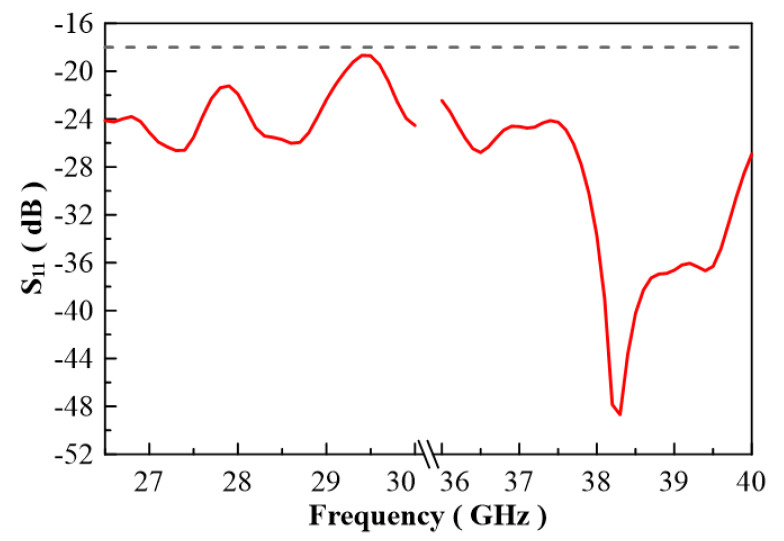
Return loss of simulation result.

**Figure 9:**
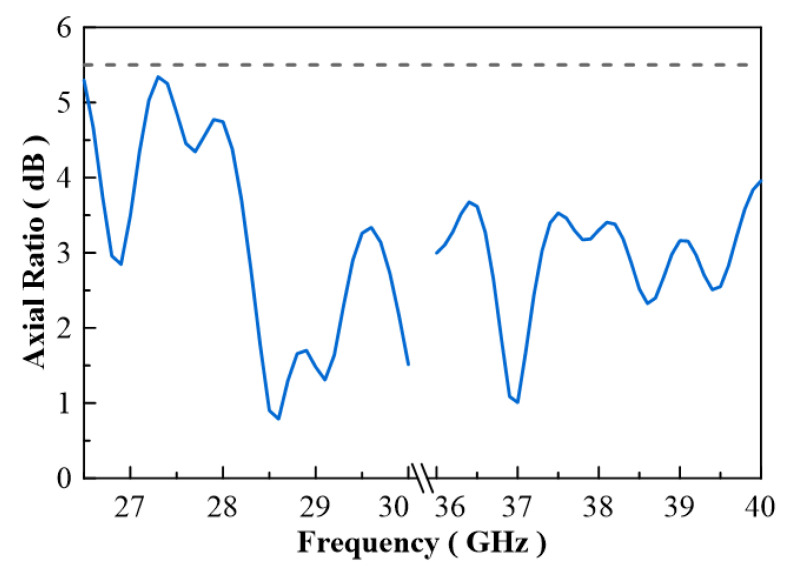
Axial ratio of simulation result.

**Figure 10:**
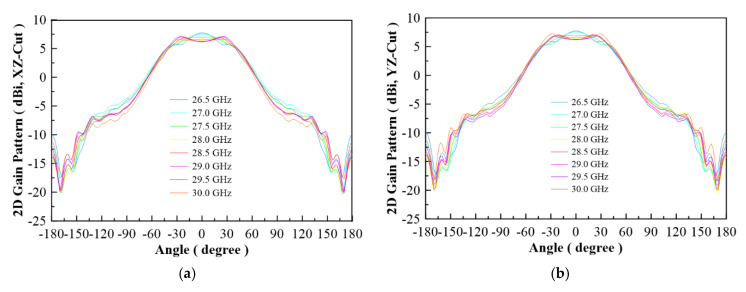

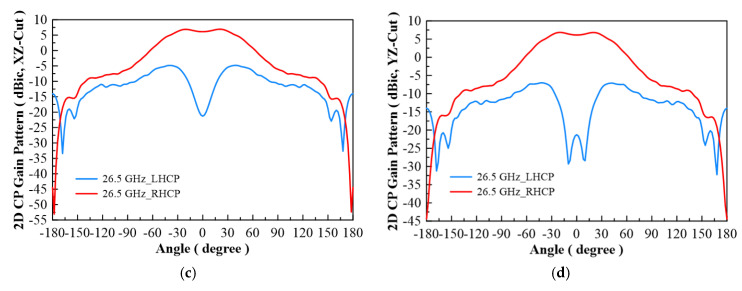
(**a**) Total gain pattern of simulation result on low band for XZ-Cut, (**b**) total gain pattern of simulation result on low band for YZ-Cut, (**c**) co-pol (RHCP) and cross-pol (LHCP) gain pattern on 28.6 GHz for XZ-Cut, and (**d**) co-pol (RHCP) and cross-pol (LHCP) gain pattern on 28.6 GHz for YZ-Cut.

**Figure 11:**
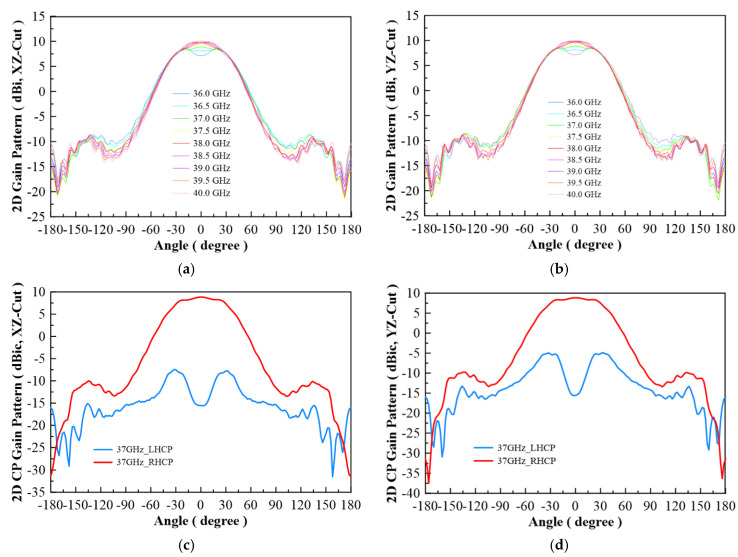
(**a**) Total gain pattern of simulation result on high band for XZ-Cut, (**b**) total gain pattern of simulation results on high band for YZ-Cut, (**c**) co-pol (RHCP) and cross-pol (LHCP) gain pattern on 37 GHz for XZ-Cut, and (**d**) co-pol (RHCP) and cross-pol (LHCP) gain pattern on 37 GHz for YZ-Cut.

**Figure 12:**
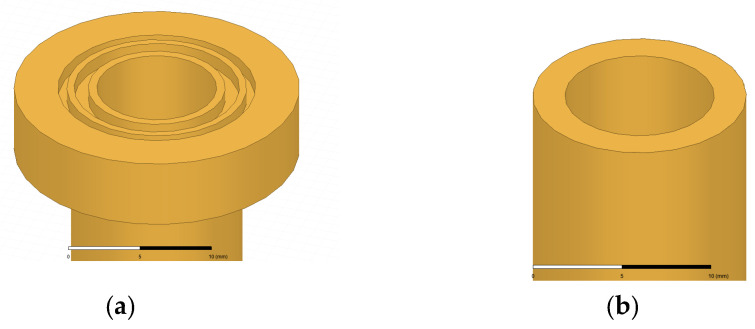
Horn antenna 3D structure (**a**) with corrugated (**b**) without corrugated.

**Figure 13:**
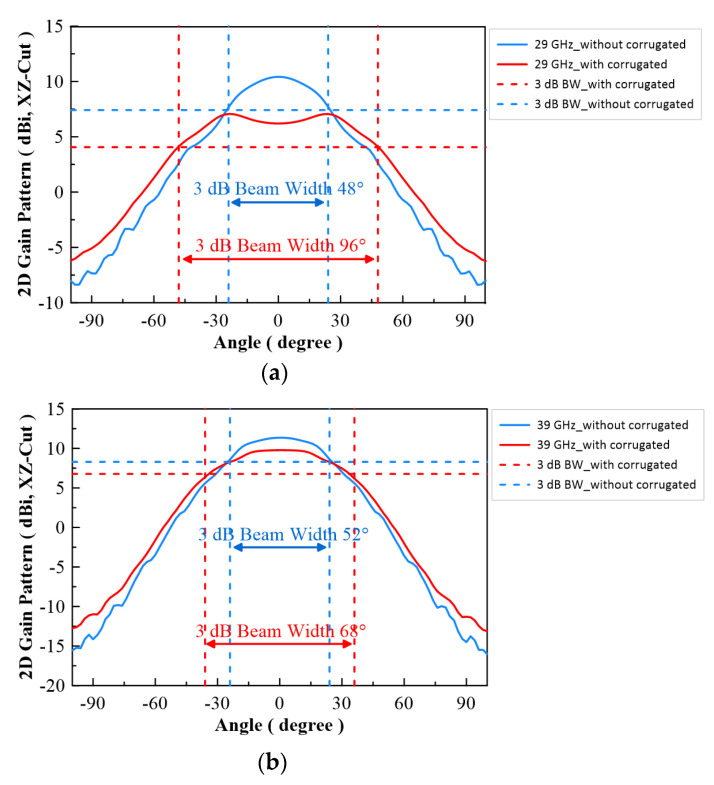
Simulation result of 2D gain pattern on XZ Cut, (**a**) Low band (29 GHz) and (**b**) High band (39 GHz).

**Figure 14:**
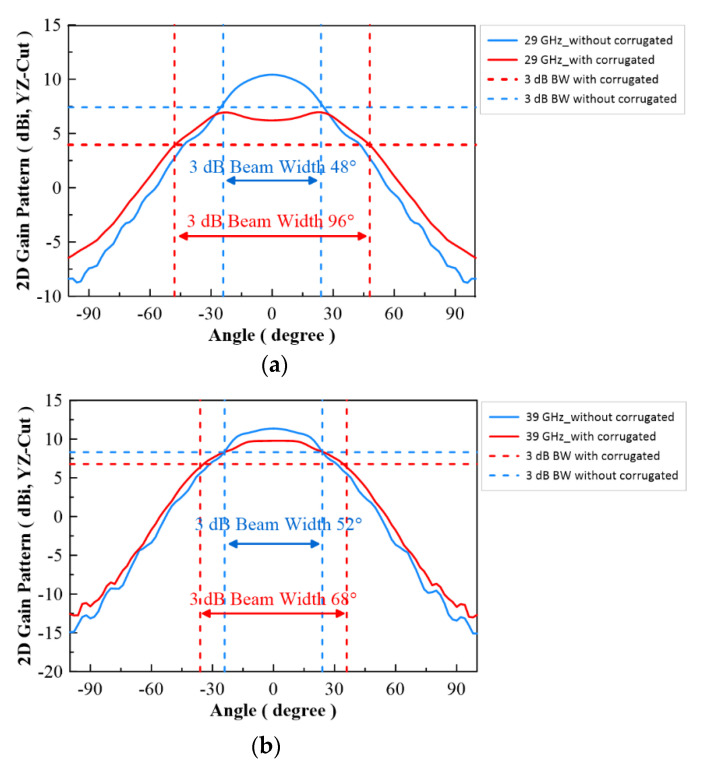
Simulation result of 2D gain pattern on YZ Cut, (**a**) Low band (29 GHz) and (**b**) High band (39 GHz).

In Figure 7, the configuration of the antenna showed the combination of a circularly polarized converter and a corrugated structure. The simulated input return loss is shown in Figure 8. Results show that |S11| < −18 dB at 26.5 GHz to 30 GHz, and 36.5 GHz to 40 GHz. This antenna bandwidth meets the specifications of bands n257 and n260. In Figure 9, the simulated axial ratio result shows that AR < 5.5 dB for low band and AR < 4 dB for high band. Figure 10 and Figure 11 show the total gain pattern and circularly polarization gain pattern of two cut planes on low band and high band, respectively. The corrugated horn antenna and the pure horn antenna are shown in Figure 12. The simulation results show that the corrugated structure improved the beam width of the horn antenna, as shown in Figure 13 and Figure 14. In low band, the beam width was increased from 48 degrees to 96 degrees; about a 100% improvement. While in high band, the beam width was increased from 52 degrees to 68 degrees; about a 30% improvement.

## 4. Antenna Manufacturing and Experimental Measurement

**Figure 15:**
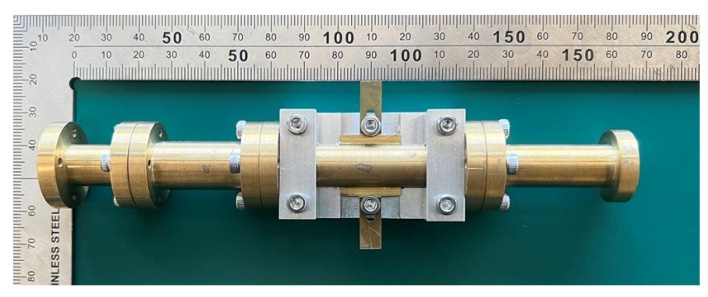
Photograph of corrugated horn antenna sample.

**Figure 16:**
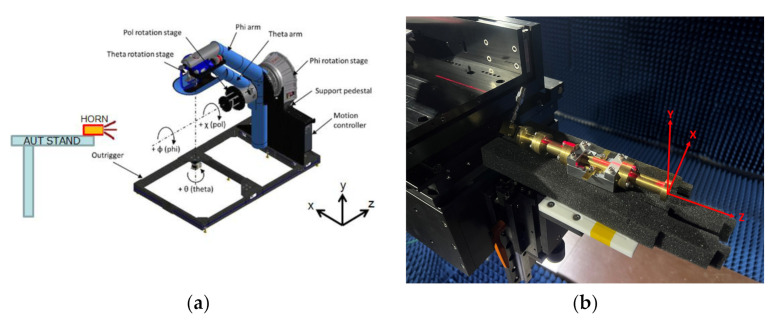
(**a**) NSI-700S-360 chamber and (**b**) measurement setting and coordinates.

**Figure 17:**
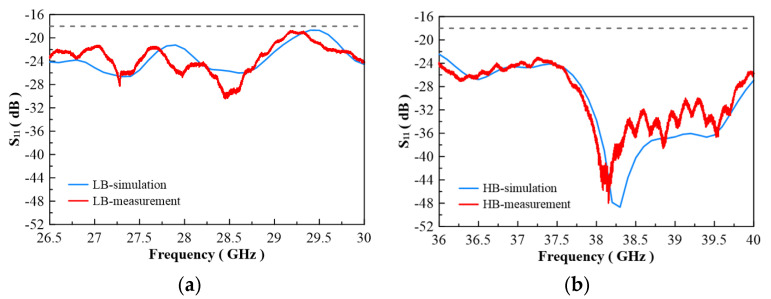
Comparison of the simulation and empirical results of the return loss at (**a**) low band and (**b**) high band.

**Figure 18:**
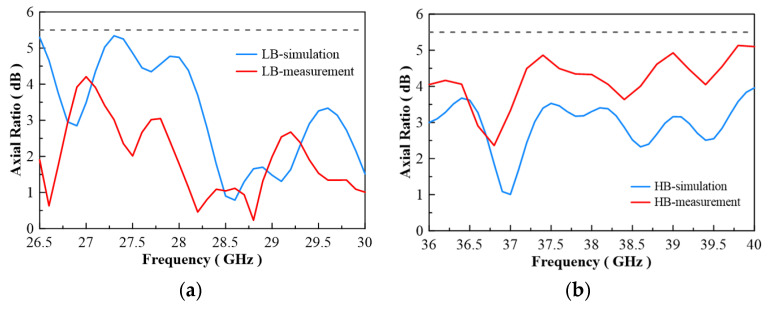
Simulation and measurement of axial ration at (**a**) low band and (**b**) high band.

The corrugated circularly polarization horn antenna is shown in Figure 15. The antenna size is 160 mm × 43 mm × 43 mm. The antenna is composed of four different components. The middle part is a circularly polarized converter, and the corrugated structure is on the right side of the photo. For the middle parts, we proposed that a reconfigurable ridge be used to optimize axial ratio by using a different depth of ridge inside the waveguide. Figure 16a shows an NSI-700S-360 antenna chamber [16]. Its measurement coordinates are shown in Figure 16b. The simulation and measurement of return loss results are shown in Figure 17. The results show consistency in simulation and measurement. Axial ratio measurement results are shown in Figure 18. The measurement results show AR < 4.5 dB on low band and <5.1 dB on high band. The difference in simulation and measurement comes from the deviation in the manufacturing process. In an optimized process, it is found that a 0.1 mm variation in ridge size will cause 1 dB of fluctuation in the axial ratio. Thus, accuracy in manufacturing the ridge is very important for this horn antenna.

**Figure 19:**
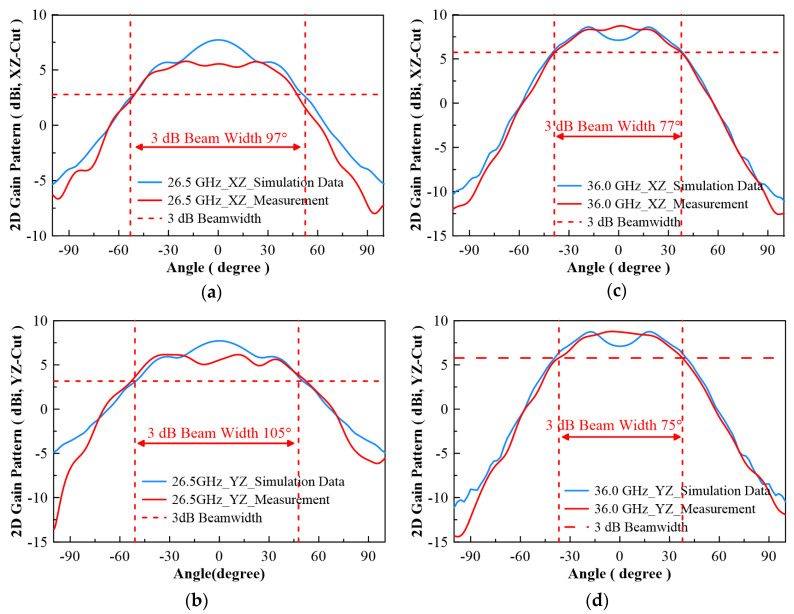
(**a**) Horn antenna gain measurement for 3 dB beam width (26.5 GHz, XZ-cut); beam width ~97 degrees, (**b**) horn antenna gain measurement for 3 dB beam width (26.5 GHz, YZ-cut); beam width ~105 degrees, (**c**) horn antenna gain measurement for 3 dB beam width (36.0 GHz, XZ-cut); beam width ~77 degrees, (**d**) horn antenna gain measurement for 3 dB beam width (36.0 GHz, YZ-cut); beam width ~75 degrees.

The measurement radiation pattern is shown in Figure 19. To attain the best beam width, 26.5 GHz and 36 GHz were selected to compare with the simulation data. The radiation pattern at 26.5 GHz shows a peak gain of 6.16 dBi and 5.78 dBi in two different cut planes, and antenna beam widths of 95 degrees and 105 degrees, respectively. The radiation pattern at 36 GHz shows peak gains of 8.74 dBi and 8.78 dBi, and antenna beam widths of 77 degrees and 75 degrees, respectively. The detailed beam width results for each frequency are listed in Table 3. The beam width ranges from 94 to 105 degrees on low band, and 60 to 77 degrees on high band. The low band beam width performance is better than the high band beam width for this design. 

**Figure 20:**
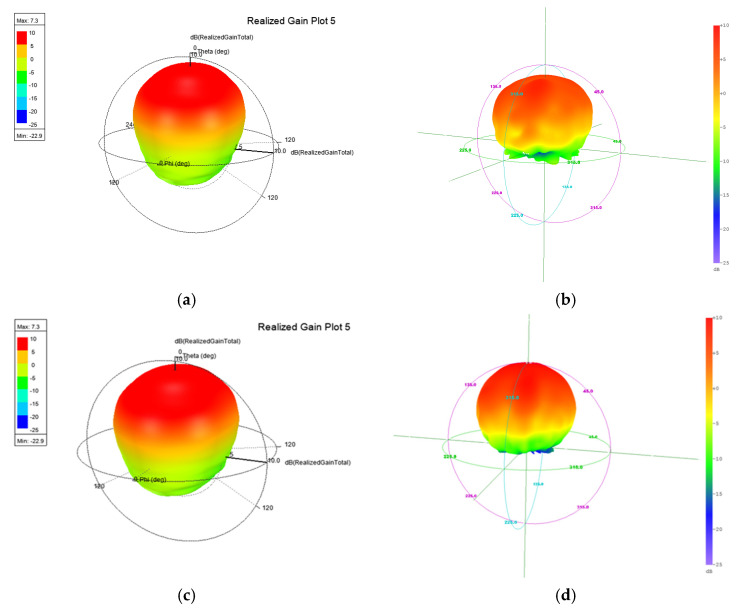
3D radiation pattern, (**a**) simulation (28 GHz), (**b**) measurement (28 GHz), (**c**) simulation (39 GHz), and (**d**) measurement (39 GHz).

**Figure 21:**
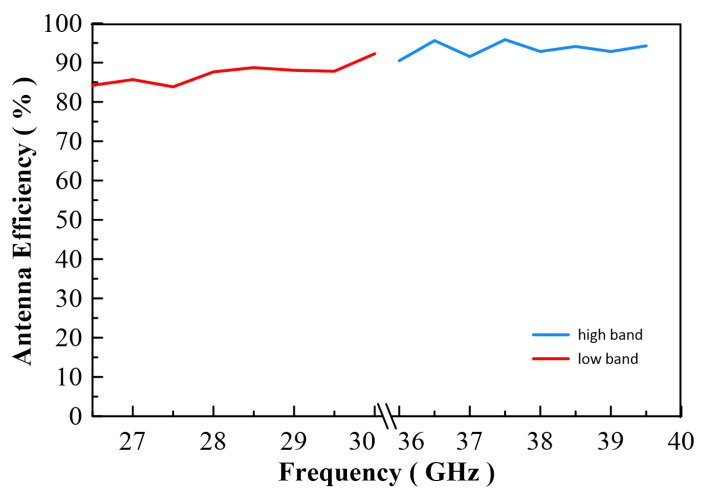
3D Radiation Efficiency.

**Figure 22:**
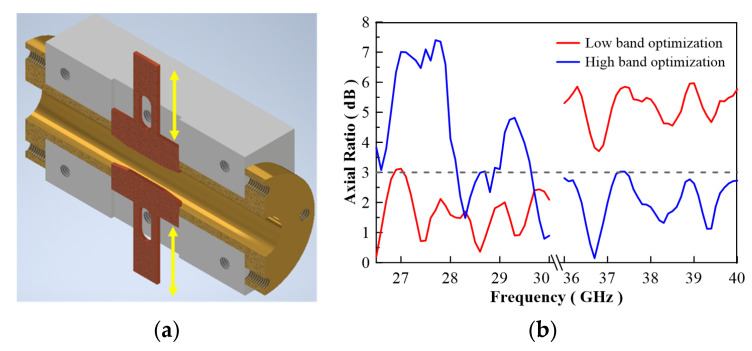
(**a**) Cross section of circularly polarization converter and (**b**) optimized axial ratio result.

Figure 20a shows a 3D simulation pattern of the antenna, and is compared to Figure 20b which was a 3D measurement pattern. The result shows the same shape as the antenna pattern. Figure 20c,d show equal performance at the 39 GHz point. Due to the reconfigurable ridge, this antenna has the capacity to optimize any band by using the tuning ridge. Figure 21 shows antenna radiation efficiency; the efficiency is about 85% to 95% on both bands. The frequency graph in Figure 22b indicates that the antenna has great axial ratio performance (AR < 3.1 dB) on the high band by pushing 0.2 mm inside the waveguide. However, this movement causes the deterioration of the low band axial ratio. This performance is reversed by pulling the ridge. This application will lead to another reconfigured design and is a future project for study. 

## 5. Conclusions

**Figure 23:**
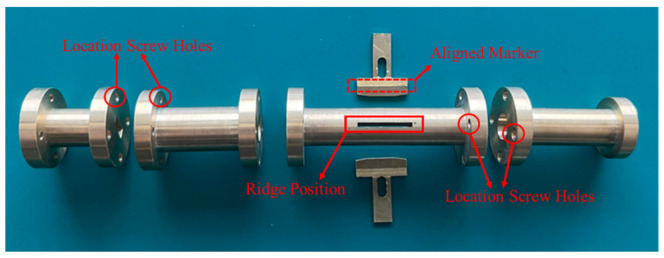
Antenna parts breakdown illustration.

The dual-band corrugated circularly polarized horn antenna has been successfully designed, fabricated, and analyzed. By using a circularly polarization converter, the antenna achieved an axial ratio of 3~5 dB. The antenna has a broad beam width of 105 degrees and 77 degrees by using two different gap sizes of corrugated structure. The resulting measurement of the antenna satisfied the requirements of the 5G indoor coverage environment. The novelty of the proposed antenna is a multi-requested feature for the application of 5G communications, e.g., dual-band, circularly polarization, and broad beam width. The existing work developed a dual-band horn antenna suitable for satellite communication, but not for 5G communications. The fabrication complexity of horn antennas for satellite application is high with high manufacturing costs. The proposed antenna is easy to manufacture and assemble. Figure 23 illustrates the parts of the antenna made using conventional CNC machining, and wire electrical discharge machining, with a tolerance of +/−0.01 mm. It is easy to assemble this antenna engineering design since there are aligned markers on the ridge structure as well as markers for the location of the screw holes on each section. The material used to make this antenna is brass in order for the antenna to withstand high power input flow. Depending on its application, the material can be changed to one with a low loss metal or coating surface. Even in the 5G products, the antenna can be combined with the thermal sink on a one-piece molding [6]. This design reduces the cost of the antenna’s production.

In previous research, various horn antennas have been studied with different features, such as broad beam width [10] and circularly polarization [8,9,17,18,19]. In [20], metasurface was designed to create a circular polarization antenna. The concept of metasurface antenna is phase manipulation of electromagnetic wave. The material of antenna is a PCB or a plastic plane that is easy to fabricate. The height of the metasurface antenna is low, but the bandwidth was designed to meet a specific frequency band. This reconfigurable antenna technology can also be used to mimic the characteristics of circular polarization. In the study in [21], it was found that antenna can have three different polarizations by rotating the SSPP element. The performance of the antenna is determined by its broadband bandwidth and good axial ratio. For it to be high performing, the antenna structure will be complex and expensive. 

As listed in Table 4, the proposed antenna has a better beam width and a good axial ratio on both bands compared to other previously proposed antenna designs. Due to these improvements in performance, the antenna proposed in this study can effectively use 5G in an indoor coverage environment. In future works, the authors aim to study a reconfigurable ridge that is combined with a stepper motor and switch circuit to allow the antenna a better axial ratio on a specific scenario and frequency band. The current design in this study is a single antenna design that is designed to be suitable for 5G repeater products. Future work will include the implementation of a multi-antenna MIMO system, which will be another key technological research.

## Figures and Tables

**Table 1:** Parameters of circularly polarized converter.

Parameters	Values
A1	17 mm
A2	9 mm
H	1.46 mm
θ	8.5 degrees
D	1.50 mm

**Table 2:** Parameters of the corrugated structure.

Parameters	Values (mm)
R1	10
R2	4.2
H1	1
H2	5
L1	0.6
L2	0.5
W1	1
W2	0.63

**Table 3:** Maximum scan angle with different antenna spacing.

3 dB Beam width	XZ-Cut (Degrees)	YZ-Cut (Degrees)
26.5 GHz	97	105
27.0 GHz	101	101
27.5 GHz	103	100
28.0 GHz	98	104
28.5 GHz	100	102
29.0 GHz	101	95
29.5 GHz	95	94
30.0 GHz	94	97
36.0 GHz	77	75
36.5 GHz	74	68
37.0 GHz	70	70
37.5 GHz	69	67
38.0 GHz	68	66
38.5 GHz	67	64
39.0 GHz	65	60
39.5 GHz	65	62
40.0 GHz	63	60

**Table 4:** Comparisons of antenna performance with previously proposed antennas.

	Frequency	3 dB Beam Width	Axial Ratio	Peak Gain	Fabrication Complexity
This work	24~30 GHz 37~40 GHz	105/77	<4.5 dB <5.1 dB	6.1/8.7	easy
[10]	55~62.5 GHz	112.37	NA	7.32	easy
[8]	50~75 GHz	30–60	<3.2 dB	12.21~12.56	easy
[17]	19.6–21.2 GHz 29.4–31 GHz	23 19	N/A	N/A	mid
[9]	75–110 GHz	58.3~76	<3 dB	6.7~9.8	easy
[18]	1.0~1.7 GHz	98.6	N/A	3.4~2.5	hard
[19]	1.19~1.22 GHz 1.551~1.577 GHz	94/90	<3 dB	6~6.4	easy
[20]	10.8~11.3 GHz	N/A	<3 dB	19.8 dBic	mid
[21]	8.45–11.5 GHz	N/A	<3 dB	N/A	hard
